# circ-0007707/miR-429/PDGFD Pathway Regulates the Progression of Gastric Cancer by Modulating the Immune-Gene Signature

**DOI:** 10.1155/2022/2214686

**Published:** 2022-04-25

**Authors:** Yang Yang, Weibiao Kang, Yu Yuan, Chen Duan, Wei Chen, Changjun Yu

**Affiliations:** ^1^Department of General Surgery, The First Affiliated Hospital of Anhui Medical University, 81, Meishan Road, Shushan District, Hefei City, 230000 Anhui Province, China; ^2^Anhui Medical University, 81, Meishan Road, Shushan District, Hefei City, 230000 Anhui Province, China

## Abstract

**Background:**

Immunotherapy is an important treatment modality for gastric cancer, therefore, it is crucial to understand the regulators of the tumor microenvironment in gastric cancer. Numerous studies have shown that noncoding RNAs have a critical status in the tumor progression, and the influence of competing endogenous RNA (ceRNA) networks on gastric adenocarcinoma has been widely discussed over the years, but the connection between ceRNA networks and the immune microenvironment of cancer is unclear. This study was aimed at exploring how ceRNA networks influence the prognosis of patients with gastric cancer by modulating the tumor microenvironment.

**Methods:**

The Gene Expression Omnibus was analyzed to obtain differential expression matrixes of the noncoding RNAs (circular RNAs (circRNAs), microRNAs (miRNAs)), and mRNAs. The Circular RNA Interactome web tool and TargetScan were applied to determine the miRNA binding sites of the circRNAs and miRNA target genes. The Cancer Genome Atlas provided prognostic genes for gastric cancer, and Cytoscape created the ceRNA networks. Real-time quantitative reverse transcription polymerase chain reaction and western blot assay were adopted to find out how the ceRNA network regulates the expression of the hub gene. Additionally, the TISIDB and TIMER databases were used to assess the link between the hub gene and immunotherapy, with TISIDB providing the immune genes that are coexpressed with the hub gene. Furthermore, the immune-gene signature was constructed by using Cox regression analysis. Moreover, the nomogram, which could predict the prognostic role of gastric cancer patients was created on the basis of the immune-gene signature.

**Results:**

In gastric cancer, the circ-0007707/miR-429/PDGFD pathway had a differential expression. The results demonstrated that the pathway could regulate the progression and immune microenvironment of gastric cancer by modulating the immune-gene signature, which included two immune genes (*TAB1* and *CXCR4*). Moreover, the low-risk group patients had better survival.

**Conclusion:**

The circ-0007707/miR-429/PDGFD pathway may play a regulatory role in the progression and prognosis of gastric cancer by interfering with the tumor microenvironment, and the PDGFD-related immune-gene signature could be considered a moderator of prognostic factor for gastric cancer and to guide immunotherapy programs.

## 1. Background

Gastric carcinoma is a malignant epithelial tumor of the gastric mucosa. Due to its high incidence and ranks third among all malignant tumors in terms of mortality rate [1, 2]. Every year, approximately 7,84,000 people die from gastric cancer globally [3]. It is frequently diagnosed at an advanced stage because its early symptoms are not typical. Therefore, the outcome of radical surgery is often poor; although there are a variety of adjuvant therapies available, such as chemotherapy, targeted therapy, and immunotherapy, their effects are unsatisfactory [4, 5]. Currently, the function of human epidermal growth factor receptor 2 (HER-2) on tumors is widely being studied, and many different HER-2 monoclonal antibodies have been shown to have effects on different tumors. However, studies have shown that only 13%–22% gastric cancer patients overexpress the HER-2 protein. As a result, it cannot be used as a general treatment for gastric cancer. Meanwhile, despite the discovery of many genes and signaling pathways related to gastric cancer, and the use of immune checkpoint inhibitors in clinical practice, the mortality rate of gastric cancer remains high [6, 7]. Thus, it is necessary to find sensitive molecular markers and effective treatments for gastric cancer.

The tumor microenvironment is the basic condition that regulates the growth and death of tumor cells, and numerous immune cells and immune genes are key factors in regulating the tumor microenvironment. Therefore, it is important to find out the pathways and factors that can regulate immune cells and immune genes. Immune loci such as programmed cell death protein 1/programmed death-ligand 1 (PD-1/PD-L1) have been discovered, and the antibodies to PD-1/PD-L1 have been studied. In clinical practice, immunotherapy has also been already used in the therapy of gastric cancer [8]. But, the overall results of immunotherapy for gastric cancer, which is now commonly referred to as immuno-antibody therapy, are inconsistent, and its therapeutic efficiency in the clinical setting is low. However, immunotherapy research continues to offer great hope for the treatment of gastric cancer, with more and more studies focusing on the discovery of immune checkpoints and the development of antibodies [9].

An effective immune checkpoint is critical for the treatment of tumors, which can guide the diagnosis and treatment for patients with tumors. Immunotherapies that target PD-1 have been shown to have a significant impact on a variety of human cancers [10–12]. Additionally, a variety of circRNA/miRNA/mRNA networks have been shown to influence the occurrence and progression of gastric cancer [13, 14]. Xie et al. demonstrated that overexpression of circSHKBP1 can mediate the miR-582-3p/HUR/VEGF axis to drive the progression of gastric cancer [15]. However, few studies have looked out the regulatory mechanisms between the ceRNA network and tumor immune infiltration in gastric cancer.

In this research, we found a ceRNA network highly associated with the progression of stomach adenocarcinoma (STAD) using gene expression profiling from the Gene Expression Omnibus (GEO) database and clinical information for patients with gastric cancer from The Cancer Genome Atlas (TCGA) database. Additionally, using the TIMER and TISIDB databases, we evaluated how the hub gene *PDGFD* is involved with the immune cells and how immune genes are associated with the development of STAD. Furthermore, we also created a gene signature and a nomogram based on the immune genes highly associated with PDGFD and differentially expressed in gastric cancer to predict the prognosis of STAD. The findings of this study may reveal the mechanism by which the ceRNA network regulates tumor immunity and provides new perspectives on the prediction and immunotherapy of gastric carcinoma.

## 2. Materials and Methods

### 2.1. Public Database Collection

The circRNA expression profiles used in this study were GSE89143, the miRNA expression profiles used were GSE63121, and the mRNA expression profiles used were GSE118916. These profiles were obtained from the GEO database. These expression profiles were created using the GPL19978, GPL8786, and GPL15270 platforms. The TCGA database was also used to download clinical resources for gastric cancer. For further analysis, the original data was sorted into standardized data.

### 2.2. Prognosis-Related Immune Genes of Gastric Cancer

We obtained 349 immune genes related to gastric cancer from IMMPORT, and we used Venn analysis to find the differentially expressed immune genes. We then identified 10 immune genes that can be regulated by miRNAs, eight of which can be found in the ceRNA network in gastric cancer. Additionally, PDGFD has been shown to affect the prognosis of patients with gastric cancer.

### 2.3. PDGFD Coexpressed Genes in GC

We screened genes that can be coexpressed with PDGFD in gastric cancer cells through Cancer Cell Line Encyclopedia (CCLE), which contains 1457 cell lines [16]. The standards for significance were *R* > 0.5 and *P* < 0.01. In the end, 597 PDGFD-related expression genes in gastric cancer were discovered.

### 2.4. Functional Characteristics Research of PDGFD-Related Genes

The intention of this study was to clarify how the 597 PDGFD-related expression genes are involved in the development of gastric cancer. We used The Kyoto Encyclopedia of Genes and Genomes (KEGG) pathway analysis to understand which biological metabolic pathways the 597 PDGFD-related expression genes are involved in and Gene Ontology (GO) enrichment analysis to clarify the biological functions of these genes, which ran with the R packages. *P* < 0.05 was regarded the data significant.

### 2.5. PDGFD Can Facilitate the Development of Gastric Carcinoma

To clarify the regulatory role of the targeted gene PDGFD in gastric cancer, we compared the differential expression of PDGFD in gastric cancer tissues and normal gastric mucosal tissues from the TCGA-STAD database and the effect of high PDGFD expression on the survival time for gastric cancer patients. The results revealed that PDGFD expression was higher in tumor tissues and that patients with higher PDGFD expression had a poorer prognosis.

### 2.6. Immune Relevance of PDGFD

As an immune-related factor, the regulation of immune molecules by PDGFD in the tumor microenvironment is unclear. To further understand the immune function of PDGFD, we obtained the association of PDGFD with numerous immune cells and immune genes in gastric cancer from the TIMER and TISIDB databases.

### 2.7. Construction of the Gene Signature

Following the discovery of immune-related genes associated with gastric cancer that coexpressed with PDGFD, we used Cox regression analyses to identify immune genes linked to prognosis. Only *P* < 0.05 indicated statistical significance in the univariate Cox regression analysis. Multivariate Cox regression analysis was used to investigate genes that are significant after univariate Cox regression analysis. Then, we selected the most reliable predictors with *P* < 0.05. In this analysis, we were able to obtain the gene signature. Finally, the distribution of risk score and survival state for each patient was created based on the gene signature. Additionally, the Human Protein Atlas (https://www.proteinatlas.org/) [17] and western blot were selected to assess protein expression of the two immune-gene signatures.

### 2.8. Analysis of Clinical Data

We only reserved the patients with complete data, including age, gender, T (tumor size), N (involvement of regional lymph nodes), M (distant metastasis), stage, and overall survival (OS), due to the lack of relevant clinical information in some clinical data in the TCGA database. We used the R Studios software's “survival” and “survminer” packages to calculate these data to verify the time-dependent prognostic value of our immune-gene signature. We also used heatmaps and forest maps to visualize the correlation between immune signature and other clinical data. Furthermore, a multiple receiver operating characteristic (ROC) curve was applied to examine the prognostic reliability.

### 2.9. Building a Predictive Nomogram and Signature Verification

We created a nomogram on the basis of the immune-gene signature to predict survival for patients with gastric cancer using the “rms” package of R Studio software, which includes some clinical characters such as age, sex, stage, and risk score. Moreover, the Cox regression analyses were applied to see if the immune-gene signature can be used as an independent factor to predict the prognosis of gastric cancer patients.

### 2.10. Western Blot Assay

Using a radioimmunoprecipitation assay buffer lysis buffer mixture, containing protease inhibitors, lyse AGS, MKN45, and GES-1 cells, we collected the lysates. Further, we loaded the 30–50 *μ*g of total proteins into a sodium dodecyl-sulfate polyacrylamide gel electrophoresis (SDS-PAGE) system and then transferred it onto a polyvinylidene fluoride membrane by electrophoresis. The membranes were blocked with 5% nonfat dried milk powder dissolved in tris-buffered saline Tween-20 buffer for 1 hour at room temperature. Primary antibodies against rabbit anti-PDGFD (sc-137030,1: 1000, Santa Cruz) and rabbit anti-*α*-tubulin (sc-8035, 1: 1000, Santa Cruz) were added at 4°C and incubated overnight, followed by a 1-hour incubation at room temperature with a secondary antibody against goat anti-rabbit immunoglobulin G (IgG) (ab6721, 1: 20000, Abcam). The protein bands were detected using enhanced chemiluminescence reagents (Boster Biological Technology, Wuhan, China). Finally, the gray values of protein bands were processed using ImageJ analysis software (NIH, Bethesda, MA, USA).

### 2.11. Real-Time Quantitative Reverse Transcription Polymerase Chain Reaction (qRT-PCR)

RNA extraction is as follows: (1) laid AGS cells flat on a six-well plate, and the full-grown cells in the six-well plate were washed twice by phosphate buffered saline, and 1 mL of TRIzol lysate was added to each well to lyse the cells, shaken vigorously and placed on ice for 10 minutes; (2) 200 *μ*L of chloroform per well, shaken and mixed and placed on ice for 5 minutes; (3) 12,000*g*, centrifuged at 4°C for 15 minutes; (4) the supernatant was removed to a new 1.5 mL tube, and an equal amount of isopropanol was added, mixed, and placed on ice for 10 minutes (5) centrifuge at 12,000*g* for 10 minutes at 4°C; (6) discard the supernatant and wash the precipitate twice with 75% ethanol prepared with diethyl pyrocarbonate (DEPC) water; and (7) dissolve the precipitate in DEPC water prewarmed at 70°C, measure the concentration with NanoDrop2000, and store at -20°C for backup. Reverse transcription of cDNA is as follows: according to Toyobo Spun's ReverTra Ace RTFQ-PCR RT Master Mix kit instructions; (1) reaction solution preparation: 10 *μ*L system containing 2 *μ*L of 5x RT Master Mix and 1 *μ*g of RNA template, with the remainder made up with nuclease-free water; (2) reverse transcription reaction: mix the reaction solution gently and then carry out the reaction at the following temperatures: 37°C for 15 minutes followed by 98°C for 5 minutes and maintained at 4°C. Fluorescence quantitative PCR reaction; (1) reaction solution preparation: 20 *μ*L system containing 10 *μ*L of THUNDERBIRD SYBR® RTFQ-PCR Mix, 6 pmol each of upstream and downstream primers, 0.4 *μ*L of 50X ROX reference dye and 1 *μ*g of cDNA, and finally complete the system with sterilised distilled water; (2) fluorescence quantification reaction: after gently stirring the reaction solution well, the reaction was carried out at the following temperatures: firstly, 95°C predenaturation for 60 seconds; then 95°C, 15 seconds, 60°C, 30 seconds, 40 cycles. Finally, the melting curve was analyzed with 95°C, 15 seconds, 60°C, 15 seconds, and 95°C, 15 seconds, and repeat the above operation three times for each sample. Finally, we calculated the relative expression of the RNA using the 2^-*ΔΔ*CT^ method.

### 2.12. Overexpression of circ-0007707 in AGS Cell

We purchased circ-0007707 overexpressing plasmids from Biomedical Company (Shanghai, China). The gastric cancer cell line AGS was laid out in 6-well plates at 2 × 10^5^ cells/well to transfection; then, the circ-0007707 overexpressing plasmids were transfected into AGS cells by using the lipo2000 kit (Invitrogen, Carlsbad, CA, USA). After the circ-0007707 overexpressing plasmids transfection, the cells were incubated for 2 days and the harvested cells were detected by QRT-PCR and western blot assay.

## 3. Results

### 3.1. Differential Expression Analysis of the circRNA, miRNA, and mRNA Microarray Datasets in Gastric Cancer

**Figure 1:**
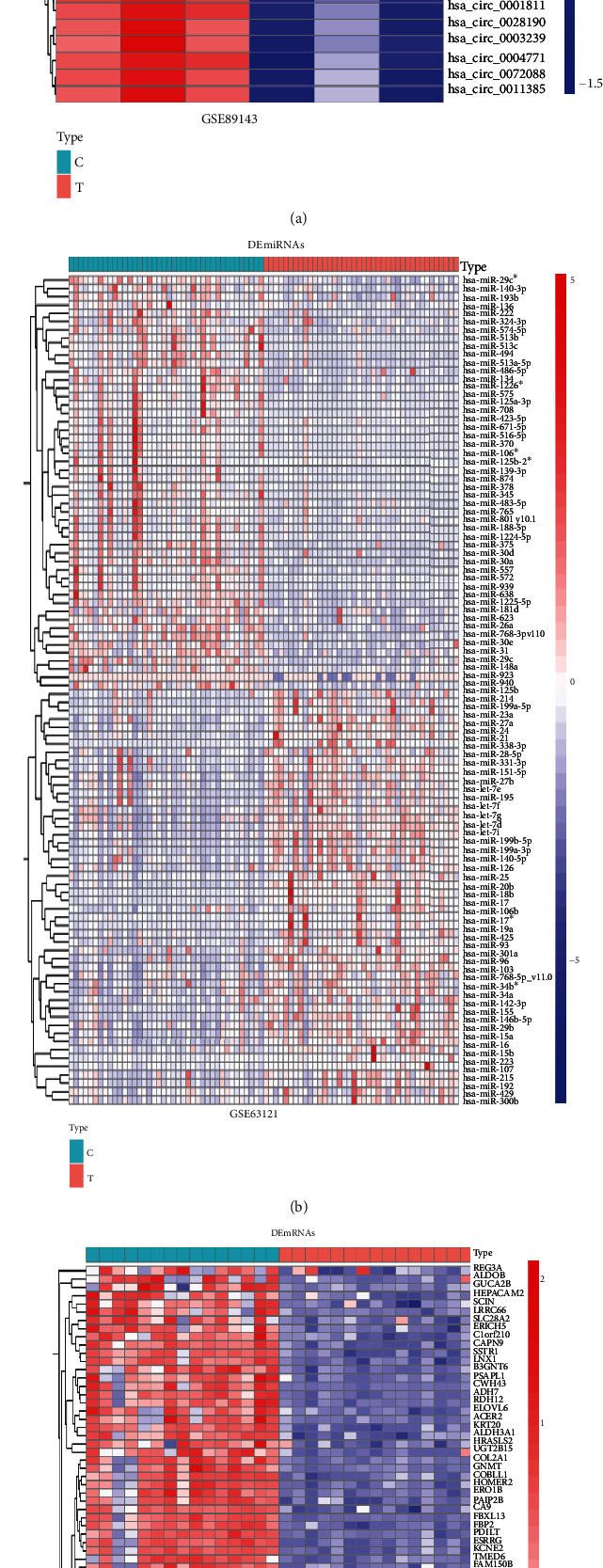
Identification of differentially expressed genes and RNAs. Hot map and volcano map of differentially expressed (a) circRNAs, (b) miRNAs, (c) mRNAs, and (d) immune genes. Red and blue/green spots represent significant upregulated and downregulated RNAs.

We found the noncoding RNAs and mRNAs with opposite expression trends in gastric cancer tissue and normal tissue using the GEO datasets. We determined 49 differentially expressed circRNAs in gastric cancer from the GSE89143 dataset, 192 differentially expressed miRNAs from in the GSE63121 dataset, and 114 differentially expressed mRNAs from GSE118916 dataset. For all the genes we obtained, only the ∣log2FC | >2 and *P* < 0.05 were indicated data significant.  Figure 1 depicts the heatmaps of the results.

### 3.2. Prediction of miRNAs and mRNAs

**Figure 2:**
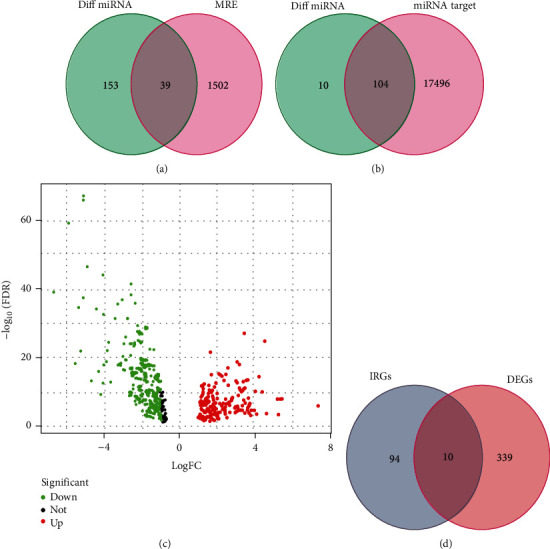
Venn diagram used to select the RNAs involved in the ceRNA network. (a) miRNAs, (b) mRNAs, and (c) immune-related mRNAs.

To further clarify the association of these noncoding RNAs and mRNAs, we also determine miRNAs and corresponding target mRNAs that can bind to differentially expressed circRNAs in gastric cancer. About 47 differentially expressed circRNAs targeting miRNAs have been predicted using the cancer-specific circRNA database. A total of 1541 miRNAs were discovered. By comparing with the miRNAs that are differentially expressed in gastric cancer, 39 target miRNAs can be found. Meanwhile, we used TargetScan to identify 17600 mRNAs linked to 39 target miRNAs, and finally, we identified 104 mRNAs that can bind to miRNA and are strongly linked to gastric cancer. The Venn diagrams for the relevant materials are shown in Figures 2(a) and 2(b).

### 3.3. The Targeting Immune Genes in Gastric Cancer

Next, we tried to see the correlation between differentially expressed circRNAs in gastric cancer and immune genes. The 2483 immune genes were collected from IMMPORT(https://www.immport.org/home), of which 349 immune genes were confirmed to be differentially expressed in gastric cancer tissues by TCGA-STAD database (Figure 2(c)). Finally, only 10 miRNA target immune genes were confirmed in gastric cancer after comparison with 104 miRNA target genes (Figure 2(d)**)**.

### 3.4. The circ-0007707/miR-429/PDGFD Pathway

**Figure 3:**
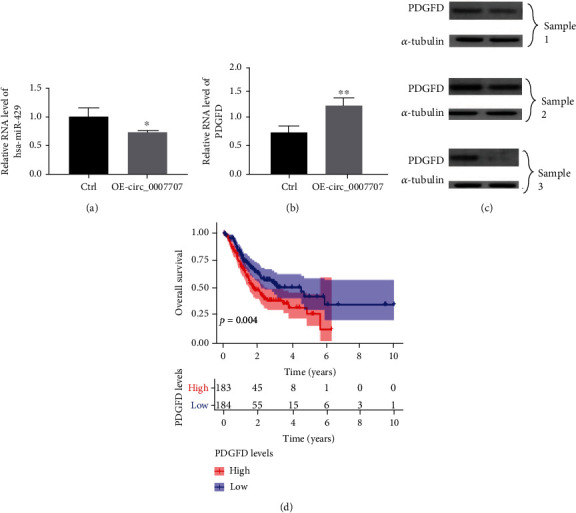
circ_0007707 regulates PDGFD expression through acting as a miR-429 sponge in GC. (a) qPCR was utilized to detect miR-429 expression upon transfection of OE-circ-0007707 in AGS cells. (b) qPCR was utilized to detect PDGFD mRNA expression upon transfection of OE-circ-0007707 in AGS cells. (c) Western blot assay was utilized to detect PDGFD protein expression in gastric cancer tissues and adjacent tissues. (d) Kaplan–Meier curve of PDGFD that significantly correlated with overall survival of gastric cancer patients.

The ceRNA network was built using Cytoscape and included eight circRNA, six miRNAs, and eight immune-mRNAs that were differentially expressed in gastric cancer (Supplementary 2). Additionally, PDGFD was the only gene involved in the prognosis of gastric cancer among the eight genes studied. Bioinformatics was used to determine the significance of circ-0007707, miR-429, and PDGFD. When circ-0007707 was overexpressed, we discovered that the expression of miR-429 dropped clearly, while the expression of PDGFD increased significantly in the AGS cell line. This suggests that circ-0007707 could absorb miR-429 to control the expression of PDGFD in gastric cancer, and the circ-0007707/miR-429/PDGFD pathway mediated the progression of gastric cancer (Figures 3(a) and 3(b)).

### 3.5. PDGFD Is Highly Expressed in Tumor Tissues and Highly Correlated with the Prognosis of Gastric Cancer Patients

circ-0007707 regulates the expression of PDGFD through the circ-0007707/miR-429/PDGFD pathway, in order to elucidate how this pathway mediates the progression and metastasis of gastric cancer and its effect on the survival time of gastric cancer patients. Western blot was selected to determine the expression level of PDGFD in normal and tumor tissues. Kaplan-Meier survival analysis was selected to analyze the intervention of PDGFD on the survival time of patients with gastric cancer. The results showed that PDGFD had higher levels of expression in cancer tissues (Figure 3(c)**)** than normal tissues. Furthermore, high PDGFD expression in patients was confirmed to be association with a worse prognosis (Figure 3(d)).

### 3.6. Biological Functions and Metabolic Pathways of PDGFD-Related Genes

**Figure 4:**
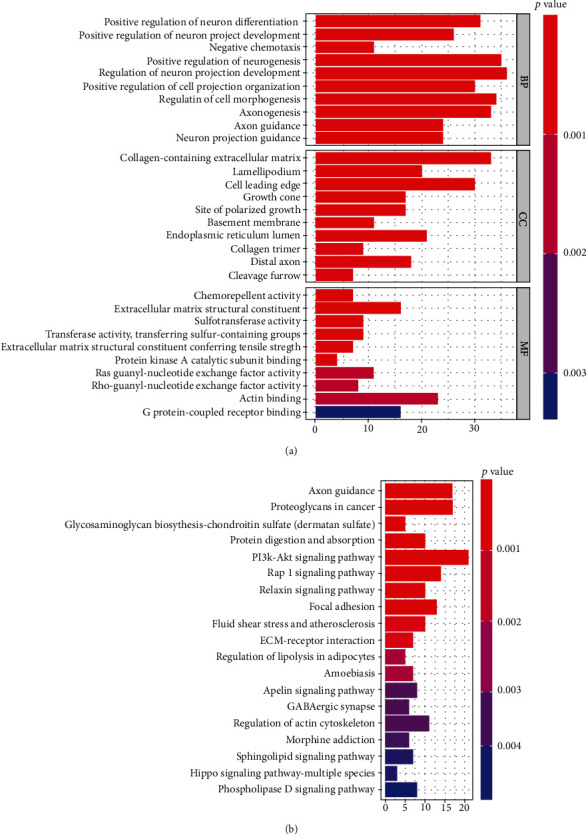
Gene Ontology (GO) and KEGG pathway enrichment analyses for the PDGFD coexpressed genes in gastric cancer. (a) Top 10 significant KEGG pathways of the PDGFD coexpressed genes in gastric cancer. (b) Top 10 enriched Gene Ontology (GO) terms of PDGFD coexpressed genes in gastric cancer. BP: biological process; CC: cellular component; MF: molecular function.

We obtained 597 genes related to PDGFD from the CCLE (sites.http://broadinstitute.org) to better understand its function, and the results are shown in Supplementary 1. The GO enrichment analysis showed that 597 related genes were enrichment in 908 GO terms (*P* < 0.05). The top 10 enriched terms in each of the three main categories are depicted in Figure 4(a), and the most enriched terms were regulation of neuron projection development, positive regulation of neurogenesis, and regulation of cell morphogenesis. These genes were related to 19 pathways in the KEGG pathway analysis (*P* < 0.05). Furthermore, the PI3K-Akt signaling pathway and Rap1 signaling pathway were the two most enriched pathways. (Figure 4(b)).

### 3.7. The Connection between Tumor Microenvironment and PDGFD

PDGFD was demonstrated to be mediated in the progression of gastric cancer as an immune-related gene. The Single-Sample Gene Set Enrichment Analysis (ssGSEA) was used to probe the link between PDGFD and immune infiltration. We found that the B cell receptor signaling pathway has a strong link to PDGFD (Supplementary 3A). Through CIBERSORT, the TCGA-STAD gene expression data were then transformed to the matrix of immune cells in gastric cancer samples. As a result, we were able to determine the proportion of immune cells in each sample. We also confirmed how PDGFD regulates immune infiltration in gastric cancer using the TIMER database. Finally, the ten immune cells (plasma cells (*P* = 0.0046), T cell CD4 memory resting (*P* = 0.00017) and activated (*P* = 9.6e − 05), T cell follicular helper (*P* = 0.0062), monocytes (*P* = 0.014), macrophages M0 (*P* = 0.016) and M1 (*P* = 4e − 05), dendritic cells (*P* = 0.014), mast cells activated (*P* = 0.029), and resting (*P* = 0.00068)) showed the high correlation with PDGFD (Supplementary 3B-K).

### 3.8. Relationship between PDGFD and Immune Genes

**Figure 5:**
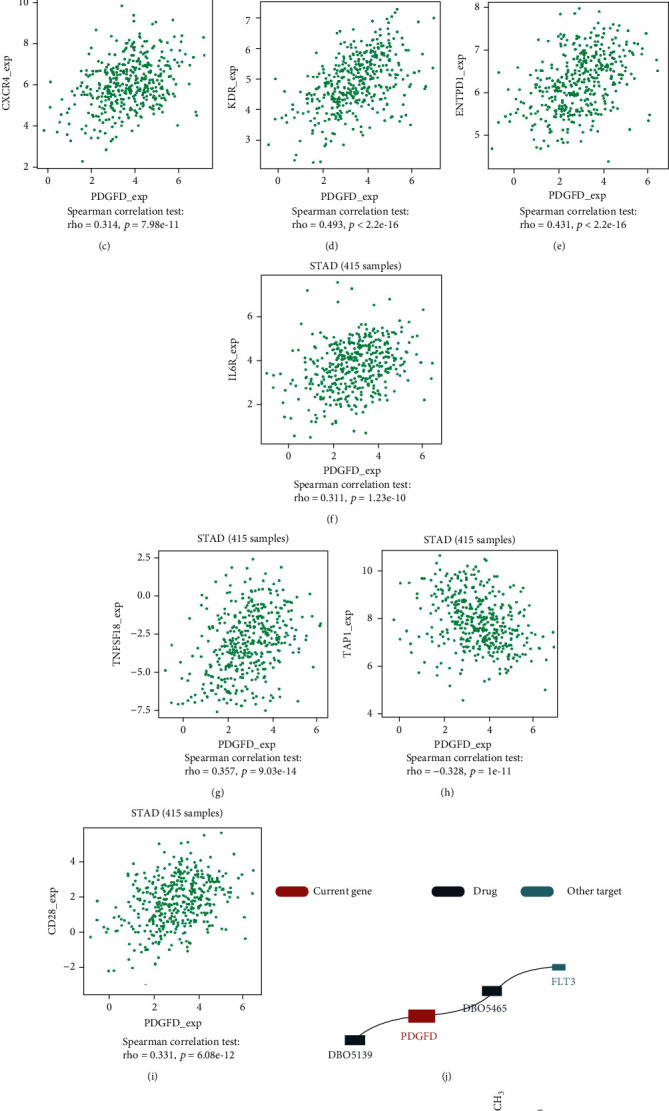
The value of PDGFD in immune therapy in gastric cancer. (a)–(h) After analyzing in TISIDB, PDGFD was significantly correlated with eight immune biomarkers in immunotherapy. (i) In TISIDB, PDGFD had the most highly expression in TGF-b dominant type of gastric cancer. (j) and (k) PDGFD was found to be the target of the type III receptor tyrosine kinases.

PDGFD was found to have a strong relationship with the immune cells. We wanted to further explore the impact of PDGFD on immunotherapy. TISIDB revealed five immune subtypes related to gastric cancer; we discovered that PDGFD has higher expression in the TGF-B subtype compared to the other four subtypes, indicating that PDGFD may act as an immunomodulatory factor in the TGF-B subtype (Figure 5(a)). Then, we looked at the relationship between PDGFD and each immune regulator to investigate the role of PDGFD in immunotherapy, obtaining immunostimulatory, immunoinhibitory, and MHC molecular data. We discovered eight molecular correlates with PDGFD after screening (∣*R* | >0.3) (Figures 5(b)–5(i). Furthermore, we discovered that PDGFD functions as a type III receptor tyrosine kinase from TISIDB (Figures 5(j) and 5(k)). Cancer cells have been shown to activate tyrosine kinases, which speed up tumor growth. It has opened up new avenues for research into the effects of PDGFD on gastric cancer immunotherapy.

### 3.9. Screening Immune Genes Related to Prognosis and Construction of Gene Signature

**Figure 6:**
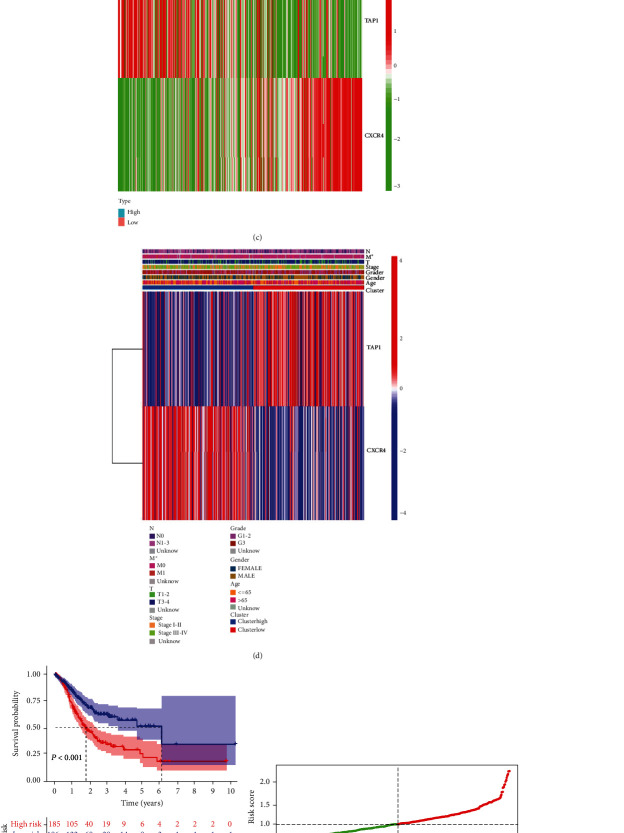
Construction of gene signature and characteristics of the two-gene signature in TCGA STAD dataset. (a) and (b) Univariance and multivariance Cox regression to identify immune-gene signature. (c) Heatmap demonstrating the distribution of the two immune-related gene expression in the TCGA cohort. (d) and (e) Distribution of risk score and patient survival time, the black dotted line is the optimal cut-of value for dividing patients into low-risk and high-risk groups. (f) Kaplan-Meier survival curves of the two gene signature of patients with STAD in the TCGA cohort. (g) Heatmap of the immune-gene signature with other clinical parameters.

The clinical information used in this study is illustrated in Table 1. We chose eight immunoregulatory genes from TISIDB for statistical analysis because they had a strong relationship with PDGFD. First, the genes (*CXCR4, TNFSF18,* and *TAP1*) were validated using univariate Cox regression analysis. Then, we chose three genes (*CXCR4, TNFSF18,* and *TAP1*) that were all linked to the length of survival for patients with stomach cancer (Figure 6(a)). The results of the multivariate Cox regression analysis **(**Figure 6(b)**)** reflected that CXCR4 and TAP1 are part of the gene signature. The coefficients of the two selected genes were determined using multivariate Cox regression analysis.

### 3.10. The Impact of Immune-Gene Signature on Clinical Prognosis

Further, we tried to make definite the clinical value of the immune-gene signature. Patients in the TCGA-STAD datasets, which were selected to evaluate the clinical value of the immune-gene signature, were split into two risk groups. According to the median based on risk score, CXCR4 and TAP1 had opposing expression trends in the high-risk group and the low-risk group (Figure 6(c)). In this cohort, we found significant differences in tumor size and the presence of distant metastasis between the two risk groups (Figure 6(d)). Furthermore, the TCGA cohort's survival analysis revealed an obvious difference from the two risk groups (Figure 6(e)), indicating that as risk increases, so does the patient's mortality rate (Figures 6(f) and 6(g)). Furthermore, the ROC curve plot (Supplementary 4) revealed that the immune-gene signature has a clear worth in analyzing the prognosis of patients with gastric cancer. In this ROC curve plot, the AUC of the risk score was 0.639, it had a higher reliability than other clinical indicators (age, gender, grade, and stage). And the AUC of risk+clinical was 0.718. This result demonstrated that our immune-gene signature combined with these clinical indicators had a more significant effect on predicting the prognosis of gastric cancer patients.

### 3.11. The Nomogram Construction

**Figure 7:**
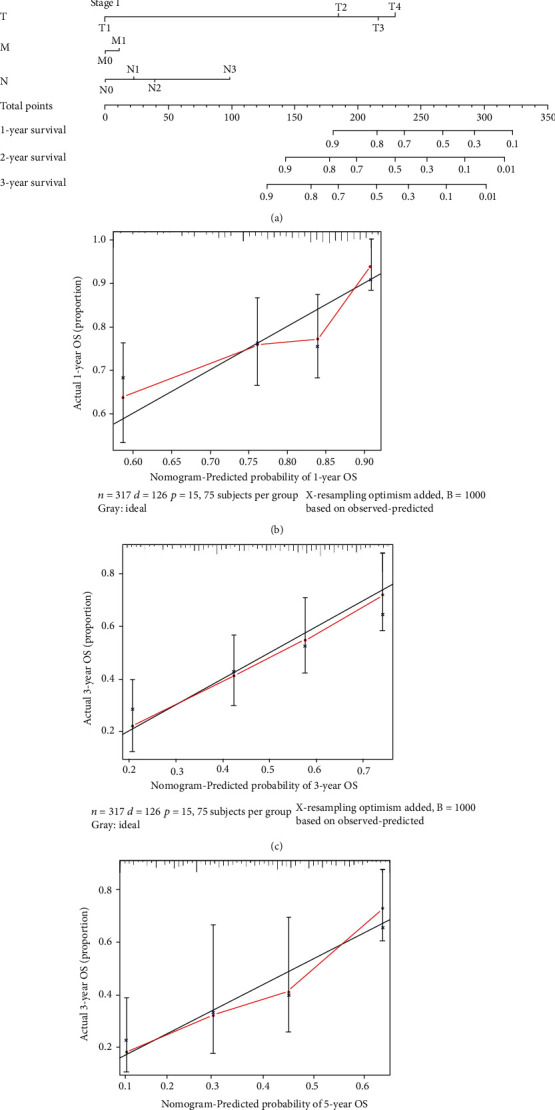
Nomogram of the gene signature. (a) Prognostic nomogram to predict the survival of STAD patients based on the TCGA cohort. (b)–(d) Calibration curves of the nomogram for predicting survival at 1, 3, and 5 years in the TCGA cohort.

**Figure 8:**
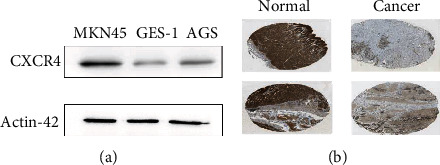
Validation of expression and alteration of the two genes in gastric cancer. (a) Western blot assay was utilized to detect CXCR4 protein in MKN45, GSE-1, and AGS cells. (b) The expression of the TAP1 in gastric cancer tumor tissue and normal tissue. Data was obtained from the Human Protein Atlas (https://www.proteinatlas.org/).

The nomogram was created to analyze the 1-year, 2-year, and 3-year overall survival for patients with gastric cancer **(**Figures 7(a)**)**. The C-index was 0.776 (SE = 0.028), indicating that it is possible to predict the survival status of patients with gastric cancer. As shown in Figure 6(a), the immune-gene signature had a better predictive effect on the prognosis of gastric cancer than stage and grade. The calibration curves further demonstrated that the 1-, 2-, and 3-year survival rates predicted by the nomogram were in line with the actual survival rates of gastric cancer over a certain time period **(**Figures 7(b)–7(d)**)**. Furthermore, the Cox regression analysis revealed that the gene signature had the potential to be used as a relatively independent prognostic factor for gastric cancer (Supplementary 5A, B). The expression of CXCR4 and TAP1 in gastric cancer was shown in Figures 8(a) and 8(b). The experimental results show that CXCR4 expression was higher in gastric cancer cells, while TAB1 expression was higher in normal gastric tissues than in gastric cancer tissues. It also further demonstrated the reliability of immune-gene signature.

## 4. Discussion

As a highly malignant tumor with a poor prognosis, gastric cancer accounts for the highest mortality rates of all cancers. Its early detection and treatment are still challenging issues. Many researchers have recently pointed out that the ceRNA network plays many important roles in the development of cancers [18–20]. Lu et al. confirmed that the circ-RanGAP1/miR-877-3p/VEGFA pathway can promote invasion and metastasis of gastric cancer [21]. According to Peng et al., the circCUL2/miR-142-3p/ROCK2 pathway can regulate gastric cancer cisplatin resistance [22]. Luo et al. showed that the circCCDC9/miR-6792-3p/CAV1 pathway could restrain the development of gastric cancer [23]. We make sure that more circRNA/miRNA/mRNA pathways need to be identified to treat cancer.

Additionally, the tumor microenvironment is important in tumorigenesis. More studies are now beginning to focus on the tumor microenvironment to better understand tumor treatment. Although there are many current studies on gastric cancer immunity, which make us understand the importance of immune regulation in the occurrence and development of gastric cancer. But there has been little research on how immune effects in gastric cancer are regulated by ceRNA networks. We investigated whether the circRNA-miRNA-mRNA pathway can regulate the expression of immune genes in cancers to achieve tumor immunotherapy effects [24]. In this study, we used the GEO database, TCGA database, and IMMPORT database to construct the circ-0007707/miR-429/PDGFD pathway, which can regulate downstream immune genes by PDGFD. RT-PCR revealed that expression of circ-0007707 and miR-429 in gastric cancer cells and normal mucosal cells was significantly different, and western blot revealed that PDGFD is differentially expressed in gastric cancer tissues. In this study, we identified PDGFD, a targeted gene of circ-0007707/miR-429/PDGFD pathway, which could regulate 10 important immune cells in the tumor microenvironment of gastric cancer and was also involved in the expression of several immune genes. This demonstrated that circ-0007707/miR-429/PDGFD pathway could mediate the development of gastric cancer by regulating the tumor microenvironment of gastric cancer. Furthermore, gastric carcinoma patients with a high level of PDGFD expression will have a poor prognosis. Liu et al. found that PDGFD can induce proliferation and invasion in breast cancer, giving patients a poor prognosis [25]. Our study also found that in the gastric cancer cell that overexpressed circ-0007707, the expression of miR-429 decreased while the expression of PDGFD increased. However, further research should clarify the molecular mechanism of the circ-0007707/miR-429/PDGFD pathway, and interestingly, the expression of PDGFD mRNA and protein in gastric cancer shows two opposing trends. We evaluated how PDGFD mRNA undergoes certain chemical modifications during translation in gastric cancer, resulting in higher levels of PDGFD protein in cancer tissues. However, at a later stage, the specific regulation mechanism will need to be verified.

The coexpression genes of PDGFD were proved to be mediated in many tumor pathways, including the PI3K-Akt signaling pathway and proteoglycans. According to the results of GSEA, PDGFD is involved in the B cell pathway. Sun et al. demonstrated that PDGFD activated natural killer cells that play important clinical roles in the immune surveillance of LGG [26]. As a result, we conducted immune correlation research on PDGFD and discovered that TIMER and TISIDB found PDGFD to be highly correlated with twelve immune cells and eight immune regulatory genes. Finally, we used the eight immunoregulatory genes coexpressed with PDGFD in gastric cancer and the TCGA dataset to create an immune-associated signature made up of two genes (*CXCR4* and *TAP1*). Another study also showed that PDGFD can activate CXCR4 to promote lymphatic metastasis in breast cancer [25]. There were 384 cases of gastric cancer in TCGA-STAD database. We utilized the univariate and multivariate Cox regression analyses to demonstrate that the signature could influence the prognosis of patients with gastric cancer [27–29]. We discovered significant differences in tumor size and OS between the two groups after splitting the patients into two risk groups based on gene signature. We built a predicted nomogram model based on the signature, and the nomogram could aid in the selection of a treatment strategy for patients with gastric cancer. Our study revealed novel protein-protein interactions in gastric cancer that could be used for immunotherapy (PDGFD-CXCR4; PDGFD-TAP1), but more research is still needed to understand the molecular action processes in detail.

In conclusion, this research showed that a ceRNA network controls immune-gene signature in gastric cancer. Unlike previous studies, our research found that the circ-0007707/miR-429/PDGFD pathway influenced the development of gastric cancer and that the immune gene *PDGFD* not only regulates immune infiltration but also regulates the downstream immune genes to influence the length of survival for patients with gastric cancer. However, the results of this research are based on bioinformatics and some simple biological experiments. Research into specific pathways and immune regulation will have to wait until later.

## 5. Conclusions

In this study, we constructed the circ-0007707/miR-429/PDGFD pathway which could mediate the development of gastric cancer and patients' prognosis. The increased expression of PDGFD was linked to tumor progression, immune infiltration, and prognosis of patients with gastric cancer. The signature constructed based to the downstream immune gene of PDGFD could be selected as an important prognostic factor in gastric cancer. In conclusion, circ-0007707/miR-429/PDGFD regulates immune infiltration, and immunomodulatory genes may be the key mechanism and a new target for immunotherapy in gastric cancer.

## Figures and Tables

**Table 1:** Patients' characteristic.

Clinical features	Number
Survival status	
Alive	235 (61.20%)
Dead	149 (38.80%)
Age	
≥67	199 (51.82%)
<67	185 (48.18%)
Sex	
Female	142 (36.98%)
Male	242 (63.02%)
Grade	
G1	8 (2.08%)
G2	133 (34.64%)
G3	243 (63.28%)
Stage	
Stage I	51 (13.28%)
Stage II	121 (31.51%)
Stage III	170 (44.27%)
Stage IV	42 (10.94%)
T	
T1	19 (4.95%)
T2	79(20.57%)
T3	181 (47.14%)
T4	105 (27.34%)
M	
M0	357 (92.97%)
M1	27 (7.03%)
N	
N0	123 (32.03%)
N1	102 (26.56%)
N2	79 (20.57%)
N3	80 (20.83%)

## Data Availability

The raw transcriptome data of this study are available in the TCGA database (TCGA-STAD cohorts) (https://portal.gdc.cancer.gov/) and the GEO database (GSE89143, GSE63121, and GSE118916). Immunization-related information was obtained from TIMER and TISIDB. which are publicly available databases.

## Supplementary Materials

Supplementary Materials Supplementary 1. The coexpressed genes of PDGFD. Supplementary 2.The circRNA-miRNA-hub gene network based on eight circRNAs, six miRNAs, and the eight hub genes that immune genes differentially expressed in gastric cancer tissues and normal tissues. Supplementary 3. Relationship between PDGFD and immune infiltration. (A) PDGFD enriched in the B cell receptor signaling pathway by gene set enrichment analysis. (B-K) According to the TIMER database, 10 immune cells were suggested to have a significant correlation with PDGFD. Supplementary 4 ROC curves measuring the predictive value of the two-gene signature. Supplementary 5 Univariance and multivariance Cox regression analysis of the gene signature in the TCGA cohort. (A) Univariance Cox regression analysis. (B) Multivariance Cox regression analysis.
