# Supplementary material for: circ-0007707/miR-429/PDGFD Pathway Regulates the Progression of Gastric Cancer by Modulating the Immune-Gene Signature
Source: J Oncol. 2022 Apr 25;2022:2214686. doi: 10.1155/2022/2214686 (PMC9061023; doi:10.1155/2022/2214686)
Supplement: Supplementary Materials — Supplementary 1. The coexpressed genes of PDGFD. Supplementary 2.The circRNA-miRNA-hub gene network based on eight circRNAs, six miRNAs, and the eight hub genes that immune genes differentially expressed in gastric cancer tissues and normal tissues. Supplementary 3. Relationship between PDGFD and immune infiltration. (A) PDGFD enriched in the B cell receptor signaling pathway by gene set enrichment analysis. (B-K) According to the TIMER database, 10 immune cells were suggested to have a significant correlation with PDGFD. Supplementary 4 ROC curves measuring the predictive value of the two-gene signature. Supplementary 5 Univariance and multivariance Cox regression analysis of the gene signature in the TCGA cohort. (A) Univariance Cox regression analysis. (B) Multivariance Cox regression analysis. [file 2214686.f1.zip › Supplementary 1 .docx]

Supplementary 1 The co-expressed genes of PDGFD

| ***Query*** | ***Gene*** | ***cor*** | ***pvalue*** |
| --- | --- | --- | --- |
| PDGFD | DFFB | -0.567364117 | 0.00025033 |
| PDGFD | PIK3CD | 0.610515086 | 6.01E-05 |
| PDGFD | RP11-558F24.4 | 0.629631835 | 2.98E-05 |
| PDGFD | ALDH4A1 | -0.551953057 | 0.000397876 |
| PDGFD | IL22RA1 | -0.537846009 | 0.000596473 |
| PDGFD | LAPTM5 | 0.547092645 | 0.000458363 |
| PDGFD | NKAIN1 | 0.556576375 | 0.000347061 |
| PDGFD | SYNC | 0.530021932 | 0.000741043 |
| PDGFD | MFSD2A | -0.680931778 | 3.52E-06 |
| PDGFD | TMEM125 | -0.523383321 | 0.000887264 |
| PDGFD | CMPK1 | -0.521960762 | 0.000921734 |
| PDGFD | FOXD2-AS1 | -0.701558889 | 1.32E-06 |
| PDGFD | FOXD2 | -0.715665937 | 6.40E-07 |
| PDGFD | PRKAA2 | 0.623318121 | 3.77E-05 |
| PDGFD | ST6GALNAC5 | 0.633426227 | 2.58E-05 |
| PDGFD | NEXN | 0.743168752 | 1.38E-07 |
| PDGFD | DNAJB4 | 0.665046531 | 7.12E-06 |
| PDGFD | LPHN2 | 0.714006284 | 6.98E-07 |
| PDGFD | TTLL7 | 0.594985479 | 0.000102782 |
| PDGFD | LPAR3 | 0.739669188 | 1.69E-07 |
| PDGFD | MCOLN3 | 0.543654793 | 0.000505966 |
| PDGFD | DDAH1 | -0.587398495 | 0.000132312 |
| PDGFD | RP4-665J23.1 | 0.610314167 | 6.05E-05 |
| PDGFD | EPHX4 | 0.568010187 | 0.000245392 |
| PDGFD | S1PR1 | 0.520569057 | 0.000956599 |
| PDGFD | VAV3 | 0.530021932 | 0.000741043 |
| PDGFD | GSTM4 | -0.539624208 | 0.000567348 |
| PDGFD | EPS8L3 | -0.52693972 | 0.000806034 |
| PDGFD | LRIF1 | 0.523738961 | 0.000878827 |
| PDGFD | DENND2D | -0.56902377 | 0.000237819 |
| PDGFD | BCL2L15 | -0.613004565 | 5.50E-05 |
| PDGFD | NGF | 0.557910279 | 0.000333521 |
| PDGFD | SV2A | 0.596289492 | 9.84E-05 |
| PDGFD | C1orf56 | 0.541758047 | 0.000534073 |
| PDGFD | MLLT11 | 0.596763678 | 9.68E-05 |
| PDGFD | PSMD4 | 0.544366073 | 0.000495771 |
| PDGFD | BX470102.3 | -0.534763797 | 0.000650119 |
| PDGFD | SNAPIN | 0.613952938 | 5.31E-05 |
| PDGFD | ATP8B2 | 0.524568788 | 0.000859416 |
| PDGFD | BGLAP | 0.536541996 | 0.000618669 |
| PDGFD | KIRREL | 0.603639382 | 7.65E-05 |
| PDGFD | OLFML2B | 0.58336791 | 0.000150926 |
| PDGFD | RGS4 | 0.611463459 | 5.81E-05 |
| PDGFD | XCL1 | 0.573022737 | 0.000209946 |
| PDGFD | TNFSF18 | 0.555690369 | 0.000356324 |
| PDGFD | LAMC1 | 0.556220735 | 0.000350753 |
| PDGFD | HMCN1 | 0.540809674 | 0.000548641 |
| PDGFD | LHX9 | 0.603082395 | 7.79E-05 |
| PDGFD | ETNK2 | 0.544721713 | 0.000490742 |
| PDGFD | RP11-739N20.2 | -0.523501868 | 0.000884443 |
| PDGFD | KCNK2 | 0.529080461 | 0.000760383 |
| PDGFD | TGFB2 | 0.584553377 | 0.000145221 |
| PDGFD | MARK1 | 0.623081028 | 3.81E-05 |
| PDGFD | CAPN8 | -0.562859345 | 0.000287314 |
| PDGFD | TMEM63A | -0.577796219 | 0.000180536 |
| PDGFD | WNT9A | 0.593444373 | 0.000108248 |
| PDGFD | PGBD5 | 0.545077353 | 0.000485759 |
| PDGFD | GNG4 | 0.568786676 | 0.000239572 |
| PDGFD | RP11-371I1.2 | 0.552163563 | 0.000395425 |
| PDGFD | DPYSL5 | 0.619643176 | 4.32E-05 |
| PDGFD | CLIP4 | 0.521368029 | 0.000936443 |
| PDGFD | EHD3 | 0.62651888 | 3.35E-05 |
| PDGFD | EPCAM | -0.592733093 | 0.000110858 |
| PDGFD | STON1 | 0.541165314 | 0.000543137 |
| PDGFD | AC007743.1 | 0.675997961 | 4.40E-06 |
| PDGFD | CCDC85A | 0.643166986 | 1.76E-05 |
| PDGFD | SPRED2 | -0.630905105 | 2.84E-05 |
| PDGFD | ANTXR1 | 0.603995022 | 7.55E-05 |
| PDGFD | ADD2 | 0.557406201 | 0.000338581 |
| PDGFD | ATP6V1B1 | 0.701914529 | 1.29E-06 |
| PDGFD | AC064872.1 | 0.52878275 | 0.000766591 |
| PDGFD | RPIA | -0.558473121 | 0.00032795 |
| PDGFD | CHST10 | 0.578033313 | 0.000179177 |
| PDGFD | SLC9A2 | -0.681524511 | 3.42E-06 |
| PDGFD | MFSD9 | -0.521842215 | 0.000924659 |
| PDGFD | SH3RF3 | 0.527888093 | 0.000785519 |
| PDGFD | AC013268.5 | -0.550088915 | 0.000420176 |
| PDGFD | AC112229.7 | -0.524094601 | 0.000870461 |
| PDGFD | CCDC74B | 0.55882876 | 0.000324474 |
| PDGFD | MGAT5 | -0.601386996 | 8.26E-05 |
| PDGFD | KIF5C | 0.534763797 | 0.000650119 |
| PDGFD | GPD2 | -0.596170945 | 9.87E-05 |
| PDGFD | AC009299.5 | 0.576526378 | 0.000187974 |
| PDGFD | CYBRD1 | 0.589887974 | 0.000121874 |
| PDGFD | DLX1 | 0.578033313 | 0.000179177 |
| PDGFD | WIPF1 | 0.76462569 | 3.61E-08 |
| PDGFD | HOXD10 | 0.598992294 | 8.97E-05 |
| PDGFD | HOXD9 | 0.686851412 | 2.67E-06 |
| PDGFD | HOXD-AS1 | 0.547566832 | 0.000452121 |
| PDGFD | AC017048.3 | 0.668840023 | 6.04E-06 |
| PDGFD | TTC30B | 0.55989568 | 0.000314241 |
| PDGFD | OSBPL6 | 0.66064019 | 8.59E-06 |
| PDGFD | CERKL | 0.563348559 | 0.000283074 |
| PDGFD | ZNF804A | 0.591775345 | 0.000114461 |
| PDGFD | FAM171B | 0.532511411 | 0.000691992 |
| PDGFD | CALCRL | 0.620497694 | 4.19E-05 |
| PDGFD | COL5A2 | 0.703929822 | 1.17E-06 |
| PDGFD | MFSD6 | -0.591429081 | 0.00011579 |
| PDGFD | AOX1 | 0.589413788 | 0.000123803 |
| PDGFD | ZDBF2 | 0.68259143 | 3.26E-06 |
| PDGFD | OBSL1 | 0.556576375 | 0.000347061 |
| PDGFD | KCNE4 | 0.601505543 | 8.23E-05 |
| PDGFD | COL4A4 | 0.562503706 | 0.000290433 |
| PDGFD | PID1 | 0.54235078 | 0.000525144 |
| PDGFD | C2orf72 | -0.549582124 | 0.000426429 |
| PDGFD | AC106876.2 | -0.596021757 | 9.92E-05 |
| PDGFD | AGAP1 | -0.544958806 | 0.000487415 |
| PDGFD | PER2 | -0.542469327 | 0.000523374 |
| PDGFD | GPR35 | -0.550056311 | 0.000420576 |
| PDGFD | UBE2E2 | 0.556694921 | 0.000345838 |
| PDGFD | RBMS3 | 0.530929439 | 0.000722816 |
| PDGFD | SUSD5 | 0.57601802 | 0.000191028 |
| PDGFD | FBXL2 | 0.551715963 | 0.000400652 |
| PDGFD | STAC | 0.736810907 | 2.00E-07 |
| PDGFD | SCN5A | 0.573528541 | 0.000206638 |
| PDGFD | TDGF1 | -0.692925608 | 2.00E-06 |
| PDGFD | SEMA3B | -0.532511411 | 0.000691992 |
| PDGFD | CHDH | -0.528955012 | 0.000762993 |
| PDGFD | WNT5A | 0.524331694 | 0.000864923 |
| PDGFD | WNT5A-AS1 | 0.59946487 | 8.83E-05 |
| PDGFD | GPR27 | 0.695322238 | 1.79E-06 |
| PDGFD | GXYLT2 | 0.533135744 | 0.000680152 |
| PDGFD | ROBO2 | 0.781763219 | 1.12E-08 |
| PDGFD | ROBO1 | 0.560725506 | 0.000306483 |
| PDGFD | EPHA3 | 0.602338523 | 8.00E-05 |
| PDGFD | PROS1 | 0.579337325 | 0.000171864 |
| PDGFD | TMEM45A | 0.568075397 | 0.000244898 |
| PDGFD | LINC00883 | 0.534171064 | 0.000660914 |
| PDGFD | BOC | 0.606865487 | 6.83E-05 |
| PDGFD | ARHGAP31 | 0.546737005 | 0.000463095 |
| PDGFD | ADPRH | 0.577914766 | 0.000179855 |
| PDGFD | MYLK | 0.541520954 | 0.000537682 |
| PDGFD | KALRN | -0.66789165 | 6.29E-06 |
| PDGFD | MBD4 | -0.601149903 | 8.33E-05 |
| PDGFD | PPP2R3A | 0.535119437 | 0.000643718 |
| PDGFD | MRAS | 0.568431037 | 0.000242222 |
| PDGFD | PLS1 | -0.612174739 | 5.66E-05 |
| PDGFD | TRPC1 | 0.631853477 | 2.74E-05 |
| PDGFD | PLOD2 | 0.697409758 | 1.61E-06 |
| PDGFD | PLSCR4 | 0.582419538 | 0.000155635 |
| PDGFD | WWTR1 | 0.555153815 | 0.00036204 |
| PDGFD | P2RY1 | 0.651212437 | 1.27E-05 |
| PDGFD | RP11-38P22.2 | 0.69780688 | 1.58E-06 |
| PDGFD | MME | 0.52729536 | 0.000798286 |
| PDGFD | MLF1 | 0.535119437 | 0.000643718 |
| PDGFD | BCHE | 0.529910484 | 0.00074331 |
| PDGFD | ECT2 | -0.544603166 | 0.000492413 |
| PDGFD | GNB4 | 0.632683304 | 2.65E-05 |
| PDGFD | TTC14 | 0.528717919 | 0.000767949 |
| PDGFD | RP11-528A4.2 | -0.533951746 | 0.000664949 |
| PDGFD | TM4SF19 | 0.530851758 | 0.00072436 |
| PDGFD | RNF168 | -0.528125186 | 0.000780463 |
| PDGFD | SLIT2 | 0.628652719 | 3.09E-05 |
| PDGFD | SMIM20 | -0.528599373 | 0.000770437 |
| PDGFD | LIMCH1 | 0.528955012 | 0.000762993 |
| PDGFD | ATP10D | 0.587991229 | 0.000129756 |
| PDGFD | LRRC66 | -0.519708376 | 0.00097874 |
| PDGFD | KIT | 0.528393598 | 0.000774774 |
| PDGFD | IGFBP7 | 0.636595342 | 2.28E-05 |
| PDGFD | NPFFR2 | 0.657658961 | 9.74E-06 |
| PDGFD | PARM1 | 0.583842097 | 0.000148621 |
| PDGFD | FAM47E | -0.521249482 | 0.00093941 |
| PDGFD | PRDM8 | 0.550681683 | 0.000412967 |
| PDGFD | RASGEF1B | -0.543180607 | 0.000512866 |
| PDGFD | PTPN13 | 0.633987317 | 2.52E-05 |
| PDGFD | SNCA | 0.624029401 | 3.68E-05 |
| PDGFD | DDIT4L | 0.533025558 | 0.000682228 |
| PDGFD | CXXC4 | 0.593088733 | 0.000109546 |
| PDGFD | PITX2 | 0.613952938 | 5.31E-05 |
| PDGFD | TRAM1L1 | 0.64339785 | 1.74E-05 |
| PDGFD | FAT4 | 0.727757692 | 3.33E-07 |
| PDGFD | MGST2 | -0.560844053 | 0.000305389 |
| PDGFD | TBC1D9 | 0.522197855 | 0.000915908 |
| PDGFD | TRIM2 | -0.605536128 | 7.16E-05 |
| PDGFD | TLR2 | 0.567048299 | 0.000252777 |
| PDGFD | MAP9 | 0.736411595 | 2.04E-07 |
| PDGFD | GUCY1A3 | 0.540858453 | 0.000547883 |
| PDGFD | RP11-588K22.2 | 0.564858824 | 0.000270334 |
| PDGFD | PDGFC | 0.621184282 | 4.08E-05 |
| PDGFD | FSTL5 | 0.642785217 | 1.79E-05 |
| PDGFD | NPY1R | 0.69090186 | 2.21E-06 |
| PDGFD | CPE | 0.77446506 | 1.86E-08 |
| PDGFD | WDR17 | 0.672555889 | 5.13E-06 |
| PDGFD | TENM3 | 0.605773221 | 7.10E-05 |
| PDGFD | PDLIM3 | 0.553318452 | 0.000382219 |
| PDGFD | FAT1 | -0.576255113 | 0.000189598 |
| PDGFD | PLEKHG4B | 0.578863139 | 0.000174492 |
| PDGFD | RP11-54F2.1 | 0.538688579 | 0.000582511 |
| PDGFD | RP11-215G15.5 | 0.588109775 | 0.00012925 |
| PDGFD | RP11-308B16.2 | 0.675662125 | 4.46E-06 |
| PDGFD | CT49 | 0.632337615 | 2.69E-05 |
| PDGFD | BASP1 | 0.56902377 | 0.000237819 |
| PDGFD | CDH6 | 0.567482664 | 0.000249417 |
| PDGFD | ADAMTS12 | 0.541046768 | 0.000544966 |
| PDGFD | EGFLAM | 0.527140535 | 0.000801651 |
| PDGFD | GHR | 0.660495346 | 8.65E-06 |
| PDGFD | CCDC152 | 0.577605207 | 0.000181637 |
| PDGFD | CTD-2201E18.3 | 0.619524629 | 4.34E-05 |
| PDGFD | EMB | 0.555153815 | 0.00036204 |
| PDGFD | MAP1B | 0.678560845 | 3.92E-06 |
| PDGFD | SSBP2 | 0.566415744 | 0.000257741 |
| PDGFD | VCAN | 0.55219015 | 0.000395117 |
| PDGFD | LINC00491 | 0.587529248 | 0.000131744 |
| PDGFD | EFNA5 | 0.676071366 | 4.38E-06 |
| PDGFD | MCC | 0.531681585 | 0.000708012 |
| PDGFD | LOX | 0.710449886 | 8.39E-07 |
| PDGFD | REEP2 | 0.570327782 | 0.000228386 |
| PDGFD | DNAJC18 | 0.535237983 | 0.000641597 |
| PDGFD | CYSTM1 | -0.524331694 | 0.000864923 |
| PDGFD | PCDHGC3 | 0.68294707 | 3.21E-06 |
| PDGFD | NR3C1 | 0.549700671 | 0.000424959 |
| PDGFD | SPINK1 | -0.522914575 | 0.000898494 |
| PDGFD | ADRB2 | 0.534052517 | 0.000663092 |
| PDGFD | AFAP1L1 | 0.566178651 | 0.000259624 |
| PDGFD | SLC26A2 | 0.603876475 | 7.58E-05 |
| PDGFD | ARSI | 0.706656394 | 1.02E-06 |
| PDGFD | NDST1 | 0.568786676 | 0.000239572 |
| PDGFD | CCNJL | 0.578270406 | 0.000177827 |
| PDGFD | TENM2 | 0.589413788 | 0.000123803 |
| PDGFD | RANBP17 | 0.547803925 | 0.000449028 |
| PDGFD | ARL10 | 0.524568788 | 0.000859416 |
| PDGFD | DBN1 | 0.63280185 | 2.64E-05 |
| PDGFD | GFPT2 | 0.611107819 | 5.88E-05 |
| PDGFD | BTNL3 | -0.573234853 | 0.000208553 |
| PDGFD | FOXQ1 | -0.555865095 | 0.00035448 |
| PDGFD | SERPINB1 | -0.678205205 | 3.98E-06 |
| PDGFD | NHLRC1 | 0.63572237 | 2.36E-05 |
| PDGFD | SPDEF | -0.53535653 | 0.000639482 |
| PDGFD | SCUBE3 | 0.542824967 | 0.000518097 |
| PDGFD | MAPK13 | -0.539861301 | 0.000563562 |
| PDGFD | UNC5CL | -0.641100114 | 1.91E-05 |
| PDGFD | OARD1 | -0.545195899 | 0.000484108 |
| PDGFD | RP11-328M4.2 | -0.606484501 | 6.92E-05 |
| PDGFD | FOXP4 | -0.532037225 | 0.000701106 |
| PDGFD | ELOVL5 | 0.532629958 | 0.00068973 |
| PDGFD | BEND6 | 0.780260818 | 1.24E-08 |
| PDGFD | RP11-301G19.1 | 0.572230239 | 0.000215225 |
| PDGFD | CD109 | 0.552427243 | 0.000392375 |
| PDGFD | COL12A1 | 0.641455754 | 1.88E-05 |
| PDGFD | SH3BGRL2 | -0.525042974 | 0.000848496 |
| PDGFD | ELOVL4 | 0.648745294 | 1.41E-05 |
| PDGFD | ANKRD6 | 0.67381898 | 4.85E-06 |
| PDGFD | EPHA7 | 0.522211094 | 0.000915583 |
| PDGFD | LIN28B | 0.666943277 | 6.56E-06 |
| PDGFD | DSE | 0.571631795 | 0.00021929 |
| PDGFD | RNF217 | 0.646908898 | 1.52E-05 |
| PDGFD | TPD52L1 | 0.569142316 | 0.000236948 |
| PDGFD | RSPO3 | 0.550404944 | 0.000416319 |
| PDGFD | MOXD1 | 0.628178532 | 3.15E-05 |
| PDGFD | RP3-325F22.5 | -0.527532453 | 0.000793157 |
| PDGFD | IL20RA | -0.550293404 | 0.000417677 |
| PDGFD | QKI | 0.526702627 | 0.000811237 |
| PDGFD | AC060834.3 | 0.605659417 | 7.13E-05 |
| PDGFD | THSD7A | 0.607077234 | 6.78E-05 |
| PDGFD | VWDE | 0.751229922 | 8.46E-08 |
| PDGFD | AC002480.3 | 0.544495177 | 0.00049394 |
| PDGFD | FAM126A | 0.649516924 | 1.36E-05 |
| PDGFD | GPNMB | 0.569379409 | 0.000235213 |
| PDGFD | DFNA5 | 0.60897398 | 6.34E-05 |
| PDGFD | SCRN1 | 0.56831249 | 0.000243111 |
| PDGFD | EEPD1 | -0.54306206 | 0.000514605 |
| PDGFD | EPDR1 | 0.561081146 | 0.000303211 |
| PDGFD | NACAD | 0.611937646 | 5.71E-05 |
| PDGFD | IGFBP3 | 0.565230278 | 0.000267281 |
| PDGFD | TPST1 | 0.558710214 | 0.000325629 |
| PDGFD | WBSCR22 | -0.547685378 | 0.000450572 |
| PDGFD | LINC00035 | -0.542824967 | 0.000518097 |
| PDGFD | MAGI2-AS3 | 0.561155367 | 0.000302532 |
| PDGFD | GNAI1 | 0.581708258 | 0.000159253 |
| PDGFD | CACNA2D1 | 0.572935808 | 0.000210519 |
| PDGFD | SEMA3E | 0.565111731 | 0.000268252 |
| PDGFD | SEMA3D | 0.638411381 | 2.12E-05 |
| PDGFD | AC002456.2 | 0.569398307 | 0.000235075 |
| PDGFD | FAM133B | -0.673700434 | 4.87E-06 |
| PDGFD | SGCE | 0.752533935 | 7.81E-08 |
| PDGFD | PEG10 | 0.769960288 | 2.53E-08 |
| PDGFD | ORAI2 | 0.554916722 | 0.000364592 |
| PDGFD | SYPL1 | -0.518997097 | 0.000997379 |
| PDGFD | PRKAR2B | 0.603165196 | 7.77E-05 |
| PDGFD | MDFIC | 0.536186356 | 0.000624849 |
| PDGFD | CAV1 | 0.607195781 | 6.75E-05 |
| PDGFD | CADPS2 | -0.519945469 | 0.000972596 |
| PDGFD | GPR37 | 0.552992191 | 0.000385909 |
| PDGFD | CCDC136 | 0.603639382 | 7.65E-05 |
| PDGFD | FLNC | 0.554798175 | 0.000365874 |
| PDGFD | JHDM1D | -0.622014108 | 3.96E-05 |
| PDGFD | JHDM1D-AS1 | -0.583842097 | 0.000148621 |
| PDGFD | SLC37A3 | -0.564044812 | 0.000277136 |
| PDGFD | DENND2A | 0.536779089 | 0.00061458 |
| PDGFD | NDUFB2 | -0.525398614 | 0.000840386 |
| PDGFD | NUB1 | -0.555390908 | 0.000359504 |
| PDGFD | SHH | -0.546262819 | 0.000469472 |
| PDGFD | MNX1-AS2 | -0.541434499 | 0.000539004 |
| PDGFD | UBE3C | -0.529666292 | 0.000748297 |
| PDGFD | MFHAS1 | -0.550293404 | 0.000417677 |
| PDGFD | TUSC3 | 0.537371822 | 0.000604461 |
| PDGFD | LOXL2 | 0.574121274 | 0.000202821 |
| PDGFD | STC1 | 0.637188075 | 2.23E-05 |
| PDGFD | TEX15 | 0.658882107 | 9.26E-06 |
| PDGFD | NRG1 | 0.537964556 | 0.000594491 |
| PDGFD | ZNF703 | -0.571750342 | 0.000218479 |
| PDGFD | RPL29P19 | 0.539262207 | 0.000573172 |
| PDGFD | SNAI2 | 0.596170945 | 9.87E-05 |
| PDGFD | TOX | 0.546532306 | 0.000465839 |
| PDGFD | RP11-25K19.1 | 0.549300811 | 0.000429935 |
| PDGFD | CRISPLD1 | 0.636476796 | 2.29E-05 |
| PDGFD | ZFHX4 | 0.57637366 | 0.000188887 |
| PDGFD | PKIA | 0.603048541 | 7.80E-05 |
| PDGFD | ZBTB10 | 0.570920515 | 0.00022421 |
| PDGFD | MMP16 | 0.605334914 | 7.21E-05 |
| PDGFD | RP11-122A3.2 | 0.537123927 | 0.000608675 |
| PDGFD | SDC2 | 0.538083102 | 0.000592514 |
| PDGFD | FBXO43 | 0.566415744 | 0.000257741 |
| PDGFD | CTHRC1 | 0.58336791 | 0.000150926 |
| PDGFD | RIMS2 | 0.587161402 | 0.000133347 |
| PDGFD | LRP12 | 0.573172901 | 0.000208959 |
| PDGFD | RP11-395G23.3 | 0.539150022 | 0.000574988 |
| PDGFD | TRMT12 | 0.524450241 | 0.000862165 |
| PDGFD | CCAT1 | -0.521249482 | 0.00093941 |
| PDGFD | ST3GAL1 | 0.534052517 | 0.000663092 |
| PDGFD | OPLAH | -0.558117481 | 0.00033146 |
| PDGFD | DGAT1 | -0.583605004 | 0.000149769 |
| PDGFD | VLDLR | 0.523383321 | 0.000887264 |
| PDGFD | AK3 | -0.552664336 | 0.00038965 |
| PDGFD | PDCD1LG2 | 0.570269674 | 0.000228799 |
| PDGFD | PTPRD | 0.704759648 | 1.12E-06 |
| PDGFD | MPDZ | 0.671803688 | 5.30E-06 |
| PDGFD | SUGT1P | 0.548217072 | 0.000443684 |
| PDGFD | SNX18P7 | 0.524507496 | 0.000860837 |
| PDGFD | RP11-262H14.1 | 0.737478515 | 1.92E-07 |
| PDGFD | LINC00537 | 0.730044254 | 2.93E-07 |
| PDGFD | PIP5K1B | -0.697054118 | 1.64E-06 |
| PDGFD | GAS1 | 0.563689172 | 0.000280155 |
| PDGFD | RP11-276H19.2 | 0.539951793 | 0.000562123 |
| PDGFD | CTSL | 0.551597417 | 0.000402047 |
| PDGFD | SYK | -0.555628002 | 0.000356984 |
| PDGFD | C9orf152 | -0.609922353 | 6.13E-05 |
| PDGFD | LPAR1 | 0.602335369 | 8.00E-05 |
| PDGFD | BSPRY | -0.555509455 | 0.000358242 |
| PDGFD | OLFML2A | 0.628652719 | 3.09E-05 |
| PDGFD | CERCAM | 0.58514611 | 0.000142442 |
| PDGFD | TBC1D13 | 0.534052517 | 0.000663092 |
| PDGFD | FNBP1 | 0.53571217 | 0.000633174 |
| PDGFD | AIF1L | 0.564518998 | 0.000273155 |
| PDGFD | GPSM1 | 0.556932014 | 0.000343403 |
| PDGFD | LRRC26 | -0.553257069 | 0.000382911 |
| PDGFD | GATA3 | 0.520775296 | 0.000951359 |
| PDGFD | CCDC3 | 0.580912863 | 0.000163388 |
| PDGFD | CASC10 | 0.529429199 | 0.000753168 |
| PDGFD | GPR158 | 0.524568788 | 0.000859416 |
| PDGFD | OGDHL | 0.622488295 | 3.89E-05 |
| PDGFD | PCBD1 | -0.624266494 | 3.64E-05 |
| PDGFD | CHST3 | 0.552427243 | 0.000392375 |
| PDGFD | SNCG | 0.527651 | 0.000790604 |
| PDGFD | PPP1R3C | 0.651532216 | 1.26E-05 |
| PDGFD | EXOC6 | -0.527413906 | 0.000795718 |
| PDGFD | RP11-175O19.4 | -0.530733212 | 0.000726723 |
| PDGFD | DUSP5 | 0.523264775 | 0.000890092 |
| PDGFD | KCNQ1 | -0.531444491 | 0.000712649 |
| PDGFD | TRIM6 | 0.562503706 | 0.000290433 |
| PDGFD | APBB1 | 0.647738724 | 1.47E-05 |
| PDGFD | TUB | 0.673700434 | 4.87E-06 |
| PDGFD | SWAP70 | 0.538201649 | 0.000590544 |
| PDGFD | RP11-351I24.1 | 0.528362279 | 0.000775436 |
| PDGFD | ADM | 0.693853359 | 1.92E-06 |
| PDGFD | AMPD3 | 0.545907179 | 0.000474306 |
| PDGFD | PDE3B | 0.539624208 | 0.000567348 |
| PDGFD | BDNF | 0.583486457 | 0.000150347 |
| PDGFD | FJX1 | 0.606721594 | 6.87E-05 |
| PDGFD | SLC35C1 | -0.522197855 | 0.000915908 |
| PDGFD | MAPK8IP1 | 0.631023651 | 2.82E-05 |
| PDGFD | NRXN2 | 0.54235078 | 0.000525144 |
| PDGFD | CPT1A | -0.559777133 | 0.000315364 |
| PDGFD | P2RY6 | 0.555272362 | 0.00036077 |
| PDGFD | SLC36A4 | 0.558473121 | 0.00032795 |
| PDGFD | AMOTL1 | 0.548515205 | 0.000439863 |
| PDGFD | RAB39A | 0.556292003 | 0.00035001 |
| PDGFD | RDX | 0.647264538 | 1.49E-05 |
| PDGFD | COLCA1 | -0.575899473 | 0.000191747 |
| PDGFD | LAYN | 0.661766449 | 8.19E-06 |
| PDGFD | BACE1 | 0.59320728 | 0.000109111 |
| PDGFD | TMPRSS4 | -0.571039062 | 0.000223383 |
| PDGFD | ESAM | -0.552664336 | 0.00038965 |
| PDGFD | EFCAB4B | 0.540098395 | 0.000559798 |
| PDGFD | RIMKLB | 0.556220735 | 0.000350753 |
| PDGFD | TMEM52B | 0.530352709 | 0.000734353 |
| PDGFD | GABARAPL1 | 0.555272362 | 0.00036077 |
| PDGFD | RASSF8-AS1 | 0.594386468 | 0.000104876 |
| PDGFD | RASSF8 | 0.613241658 | 5.45E-05 |
| PDGFD | PTHLH | 0.534499111 | 0.00065492 |
| PDGFD | CPNE8 | 0.769723195 | 2.57E-08 |
| PDGFD | CNTN1 | 0.689822774 | 2.32E-06 |
| PDGFD | PRICKLE1 | 0.759053999 | 5.18E-08 |
| PDGFD | RP11-579D7.4 | -0.544613751 | 0.000492264 |
| PDGFD | NCKAP5L | 0.596408039 | 9.80E-05 |
| PDGFD | GPD1 | -0.553019976 | 0.000385594 |
| PDGFD | SOAT2 | 0.532037225 | 0.000701106 |
| PDGFD | MAP3K12 | 0.527058267 | 0.000803444 |
| PDGFD | ARHGEF25 | 0.620235909 | 4.23E-05 |
| PDGFD | SLC16A7 | 0.692946058 | 2.00E-06 |
| PDGFD | RASSF3 | -0.638847728 | 2.09E-05 |
| PDGFD | MSRB3 | 0.530851758 | 0.00072436 |
| PDGFD | GRIP1 | 0.542587874 | 0.00052161 |
| PDGFD | LYZ | -0.530021932 | 0.000741043 |
| PDGFD | CSRP2 | 0.613478752 | 5.40E-05 |
| PDGFD | POC1B | -0.562740799 | 0.000288351 |
| PDGFD | GALNT4 | -0.554442536 | 0.000369744 |
| PDGFD | GLT8D2 | 0.726453679 | 3.58E-07 |
| PDGFD | CHST11 | 0.67453026 | 4.70E-06 |
| PDGFD | NUAK1 | 0.731195544 | 2.75E-07 |
| PDGFD | RP11-412D9.4 | 0.537253276 | 0.000606473 |
| PDGFD | TRPV4 | 0.629638411 | 2.98E-05 |
| PDGFD | GIT2 | 0.577322033 | 0.000183282 |
| PDGFD | TCTN1 | 0.643115406 | 1.76E-05 |
| PDGFD | TRAFD1 | 0.545551539 | 0.000479185 |
| PDGFD | TESC | -0.567719757 | 0.000247601 |
| PDGFD | WDR66 | 0.573765634 | 0.000205104 |
| PDGFD | PIWIL1 | -0.603644084 | 7.64E-05 |
| PDGFD | WASF3 | 0.541639501 | 0.000535875 |
| PDGFD | NBEA | 0.621539922 | 4.03E-05 |
| PDGFD | LHFP | 0.703811275 | 1.18E-06 |
| PDGFD | PCDH9 | 0.689274926 | 2.39E-06 |
| PDGFD | KCTD12 | 0.667061823 | 6.52E-06 |
| PDGFD | SLITRK1 | 0.536819614 | 0.000613883 |
| PDGFD | SLITRK5 | 0.618361321 | 4.53E-05 |
| PDGFD | GPC6 | 0.703337089 | 1.20E-06 |
| PDGFD | FAM155A | 0.582824352 | 0.000153609 |
| PDGFD | SALL2 | 0.519945469 | 0.000972596 |
| PDGFD | SLC22A17 | 0.547922471 | 0.000447489 |
| PDGFD | PRKD1 | 0.540098395 | 0.000559798 |
| PDGFD | COCH | 0.538083102 | 0.000592514 |
| PDGFD | LINC00648 | 0.617592312 | 4.66E-05 |
| PDGFD | PYGL | 0.751822656 | 8.16E-08 |
| PDGFD | TRIM9 | 0.706893487 | 1.01E-06 |
| PDGFD | FRMD6 | 0.603520836 | 7.68E-05 |
| PDGFD | FERMT2 | 0.642048487 | 1.84E-05 |
| PDGFD | TMEM229B | -0.536897636 | 0.000612544 |
| PDGFD | PLEKHH1 | -0.538912928 | 0.000578842 |
| PDGFD | LTBP2 | 0.610633633 | 5.98E-05 |
| PDGFD | ZC2HC1C | -0.623318121 | 3.77E-05 |
| PDGFD | ZDHHC22 | 0.55280741 | 0.000388013 |
| PDGFD | FLRT2 | 0.531325945 | 0.000714978 |
| PDGFD | TTC7B | 0.531207398 | 0.000717313 |
| PDGFD | FBLN5 | 0.667417463 | 6.42E-06 |
| PDGFD | C14orf132 | 0.778969831 | 1.36E-08 |
| PDGFD | DEGS2 | -0.6415743 | 1.88E-05 |
| PDGFD | CHST14 | 0.522790588 | 0.000901485 |
| PDGFD | ITPKA | -0.636239702 | 2.31E-05 |
| PDGFD | SPATA5L1 | -0.577322033 | 0.000183282 |
| PDGFD | MYEF2 | 0.669195662 | 5.94E-06 |
| PDGFD | PYGO1 | 0.549496147 | 0.000427497 |
| PDGFD | CGNL1 | 0.624147947 | 3.66E-05 |
| PDGFD | RPS27L | -0.530021932 | 0.000741043 |
| PDGFD | APH1B | 0.544958806 | 0.000487415 |
| PDGFD | IGDCC3 | 0.559098992 | 0.000321854 |
| PDGFD | IGDCC4 | 0.5609626 | 0.000304298 |
| PDGFD | CLN6 | -0.57744058 | 0.000182592 |
| PDGFD | CSPG4 | 0.564518998 | 0.000273155 |
| PDGFD | ABHD17C | -0.655088615 | 1.08E-05 |
| PDGFD | KIAA1199 | -0.551241777 | 0.000406257 |
| PDGFD | EFTUD1 | -0.527532453 | 0.000793157 |
| PDGFD | ZNF774 | -0.546855552 | 0.000461513 |
| PDGFD | RP11-66B24.2 | 0.550701727 | 0.000412725 |
| PDGFD | VASN | 0.529073559 | 0.000760526 |
| PDGFD | EMP2 | -0.523857508 | 0.00087603 |
| PDGFD | QPRT | 0.53428961 | 0.000658743 |
| PDGFD | IRX3 | 0.648450004 | 1.42E-05 |
| PDGFD | GNAO1 | 0.525635707 | 0.000835018 |
| PDGFD | MMP15 | -0.527413906 | 0.000795718 |
| PDGFD | CMTM3 | 0.614308578 | 5.24E-05 |
| PDGFD | CDYL2 | 0.548752298 | 0.000436846 |
| PDGFD | IRF8 | -0.589413788 | 0.000123803 |
| PDGFD | FAM101B | 0.538557289 | 0.000584667 |
| PDGFD | CXCL16 | -0.560014227 | 0.000313122 |
| PDGFD | ZMYND15 | -0.550056311 | 0.000420576 |
| PDGFD | ZNF232 | -0.626044693 | 3.41E-05 |
| PDGFD | EFNB3 | 0.624147947 | 3.66E-05 |
| PDGFD | MYH10 | 0.533222691 | 0.000678517 |
| PDGFD | LINC00675 | -0.646689853 | 1.53E-05 |
| PDGFD | MEIS3P1 | 0.59384773 | 0.000106792 |
| PDGFD | CTD-3157E16.1 | 0.582513404 | 0.000155163 |
| PDGFD | CENPV | -0.580167152 | 0.000167352 |
| PDGFD | ALDH3A1 | -0.553257069 | 0.000382911 |
| PDGFD | DUSP14 | 0.614782764 | 5.16E-05 |
| PDGFD | ARHGAP23 | 0.546737005 | 0.000463095 |
| PDGFD | TTC25 | 0.52693972 | 0.000806034 |
| PDGFD | ARL4D | 0.593681466 | 0.00010739 |
| PDGFD | ETV4 | -0.604350662 | 7.46E-05 |
| PDGFD | GJC1 | 0.676071366 | 4.38E-06 |
| PDGFD | CTD-2020K17.1 | 0.582893724 | 0.000153264 |
| PDGFD | TBKBP1 | 0.527058267 | 0.000803444 |
| PDGFD | PRR15L | -0.53464525 | 0.000652266 |
| PDGFD | IGF2BP1 | 0.602809556 | 7.87E-05 |
| PDGFD | LIMD2 | 0.641337207 | 1.89E-05 |
| PDGFD | SOX9 | -0.64192994 | 1.85E-05 |
| PDGFD | MYO15B | -0.537846009 | 0.000596473 |
| PDGFD | MXRA7 | 0.61039654 | 6.03E-05 |
| PDGFD | RP11-353N14.4 | -0.561866122 | 0.000296099 |
| PDGFD | SLC26A11 | 0.600082984 | 8.64E-05 |
| PDGFD | NPTX1 | 0.602928103 | 7.84E-05 |
| PDGFD | EPB41L3 | 0.548278111 | 0.0004429 |
| PDGFD | RAB31 | 0.526465533 | 0.000816469 |
| PDGFD | TUBB6 | 0.522909135 | 0.000898625 |
| PDGFD | SPIRE1 | 0.525042974 | 0.000848496 |
| PDGFD | RP11-595B24.2 | -0.586223872 | 0.000137511 |
| PDGFD | GATA6 | -0.55147887 | 0.000403446 |
| PDGFD | RP11-627G18.1 | -0.565782806 | 0.000262796 |
| PDGFD | CABYR | 0.634817143 | 2.44E-05 |
| PDGFD | CHST9 | 0.73222731 | 2.59E-07 |
| PDGFD | FHOD3 | 0.595341119 | 0.000101557 |
| PDGFD | SETBP1 | 0.553612709 | 0.000378918 |
| PDGFD | ALPK2 | 0.577796219 | 0.000180536 |
| PDGFD | DSEL | 0.637780808 | 2.18E-05 |
| PDGFD | RP11-169F17.1 | 0.604939305 | 7.31E-05 |
| PDGFD | FSD1 | 0.531088852 | 0.000719656 |
| PDGFD | TUBB4A | 0.552427243 | 0.000392375 |
| PDGFD | CERS4 | 0.528480826 | 0.000772933 |
| PDGFD | ZNF799 | -0.579692965 | 0.000169917 |
| PDGFD | ZNF443 | -0.653191869 | 1.17E-05 |
| PDGFD | hsa-mir-1199 | -0.692786439 | 2.02E-06 |
| PDGFD | HOMER3 | 0.520656749 | 0.000954368 |
| PDGFD | PLEKHF1 | 0.584316283 | 0.000146346 |
| PDGFD | APLP1 | 0.525042974 | 0.000848496 |
| PDGFD | FBXO17 | 0.554916722 | 0.000364592 |
| PDGFD | FAM83E | -0.570209236 | 0.00022923 |
| PDGFD | SPACA4 | -0.551360323 | 0.000404849 |
| PDGFD | FUT2 | -0.587042856 | 0.000133867 |
| PDGFD | IZUMO1 | -0.523383321 | 0.000887264 |
| PDGFD | FUZ | 0.554086896 | 0.000373651 |
| PDGFD | ZNF600 | -0.546025726 | 0.00047269 |
| PDGFD | ZNF468 | -0.531918678 | 0.000703401 |
| PDGFD | ZNF888 | -0.541165314 | 0.000543137 |
| PDGFD | ZNF816 | -0.606128861 | 7.01E-05 |
| PDGFD | PTPRH | -0.536897636 | 0.000612544 |
| PDGFD | TMEM238 | -0.549463578 | 0.000427903 |
| PDGFD | AC010525.6 | -0.53393397 | 0.000665276 |
| PDGFD | SDCBP2 | -0.541402407 | 0.000539495 |
| PDGFD | CRLS1 | -0.592021814 | 0.000113524 |
| PDGFD | OVOL2 | -0.686147829 | 2.76E-06 |
| PDGFD | RALGAPA2 | -0.571750342 | 0.000218479 |
| PDGFD | FOXA2 | -0.554086896 | 0.000373651 |
| PDGFD | JPH2 | 0.570090689 | 0.000230076 |
| PDGFD | MMP9 | 0.555245913 | 0.000361053 |
| PDGFD | TFAP2C | 0.520775296 | 0.000951359 |
| PDGFD | SLCO4A1 | -0.570683422 | 0.000225872 |
| PDGFD | LINC00659 | -0.602963841 | 7.83E-05 |
| PDGFD | PTK6 | -0.625926147 | 3.42E-05 |
| PDGFD | SRMS | -0.562029519 | 0.000294637 |
| PDGFD | ZNF512B | 0.542587874 | 0.00052161 |
| PDGFD | TMPRSS15 | 0.557118626 | 0.000341498 |
| PDGFD | SIM2 | -0.629126905 | 3.04E-05 |
| PDGFD | KCNJ15 | 0.540027641 | 0.000560919 |
| PDGFD | COL6A1 | 0.522316402 | 0.000913007 |
| PDGFD | COL6A2 | 0.58550175 | 0.000140797 |
| PDGFD | TUBA3FP | 0.525523219 | 0.000837561 |
| PDGFD | LRP5L | -0.587635589 | 0.000131284 |
| PDGFD | MN1 | 0.573528541 | 0.000206638 |
| PDGFD | TTC28 | 0.668484383 | 6.13E-06 |
| PDGFD | TIMP3 | 0.578270406 | 0.000177827 |
| PDGFD | RASD2 | 0.52658408 | 0.000813849 |
| PDGFD | TCF20 | -0.531444491 | 0.000712649 |
| PDGFD | PNPLA3 | 0.55951393 | 0.000317869 |
| PDGFD | KIAA1644 | 0.535237983 | 0.000641597 |
| PDGFD | FBLN1 | 0.637188075 | 2.23E-05 |
| PDGFD | PANX2 | 0.533341237 | 0.000676294 |
| PDGFD | SRPX | 0.629956732 | 2.94E-05 |
| PDGFD | CHST7 | 0.706893487 | 1.01E-06 |
| PDGFD | FGD1 | 0.522316402 | 0.000913007 |
| PDGFD | MAGEH1 | 0.579692965 | 0.000169917 |
| PDGFD | STARD8 | 0.522909135 | 0.000898625 |
| PDGFD | RGAG4 | 0.541165314 | 0.000543137 |
| PDGFD | NAP1L2 | 0.621209819 | 4.08E-05 |
| PDGFD | CHIC1 | 0.585857389 | 0.00013917 |
| PDGFD | SLC16A2 | 0.588702508 | 0.000126748 |
| PDGFD | NAP1L3 | 0.615287264 | 5.06E-05 |
| PDGFD | ARMCX1 | 0.575459395 | 0.000194436 |
| PDGFD | ARMCX3 | 0.61774643 | 4.63E-05 |
| PDGFD | ARMCX2 | 0.568549583 | 0.000241335 |
| PDGFD | GPRASP2 | 0.534763797 | 0.000650119 |
| PDGFD | NGFRAP1 | 0.58514611 | 0.000142442 |
| PDGFD | MID2 | 0.521842215 | 0.000924659 |
| PDGFD | COL4A6 | 0.529784839 | 0.000745872 |
| PDGFD | COL4A5 | 0.560725506 | 0.000306483 |
| PDGFD | KLHL13 | 0.543654793 | 0.000505966 |
| PDGFD | SMARCA1 | 0.642522673 | 1.81E-05 |
| PDGFD | ZDHHC9 | -0.562622252 | 0.00028939 |
| PDGFD | MIR503HG | 0.616538234 | 4.84E-05 |
| PDGFD | SMIM10 | 0.565212658 | 0.000267425 |
| PDGFD | FAM127C | 0.619679905 | 4.32E-05 |
| PDGFD | FAM127A | 0.539031475 | 0.000576912 |
| PDGFD | ARHGEF6 | 0.525398614 | 0.000840386 |
| PDGFD | LDOC1 | 0.523146228 | 0.000892928 |
| PDGFD | MAGEA4 | 0.532511411 | 0.000691992 |
| PDGFD | GABRQ | 0.570344469 | 0.000228268 |
| PDGFD | CSAG4 | 0.586761347 | 0.000135109 |
| PDGFD | MAGEA12 | 0.528955012 | 0.000762993 |
| PDGFD | PDZD4 | 0.540572581 | 0.000552338 |
