# Supplementary material for: circ-0007707/miR-429/PDGFD Pathway Regulates the Progression of Gastric Cancer by Modulating the Immune-Gene Signature
Source: J Oncol. 2022 Apr 25;2022:2214686. doi: 10.1155/2022/2214686 (PMC9061023; doi:10.1155/2022/2214686)
Supplement: Supplementary Materials — Supplementary 1. The coexpressed genes of PDGFD. Supplementary 2.The circRNA-miRNA-hub gene network based on eight circRNAs, six miRNAs, and the eight hub genes that immune genes differentially expressed in gastric cancer tissues and normal tissues. Supplementary 3. Relationship between PDGFD and immune infiltration. (A) PDGFD enriched in the B cell receptor signaling pathway by gene set enrichment analysis. (B-K) According to the TIMER database, 10 immune cells were suggested to have a significant correlation with PDGFD. Supplementary 4 ROC curves measuring the predictive value of the two-gene signature. Supplementary 5 Univariance and multivariance Cox regression analysis of the gene signature in the TCGA cohort. (A) Univariance Cox regression analysis. (B) Multivariance Cox regression analysis. [file 2214686.f1.zip › Supplementary 2-5.docx]

Supplementary 2

Supplementary 3

Supplementary 4

Supplementary 5
